# Parliament and Publications

**Published:** 1910-11-19

**Authors:** 


### PARLIAMENT AND PUBLICATIONS.

### THE POLITICAL SITUATION.

During the last few days the political situation has completely changed, and it is understood that Parliament will be dissolved at an early date, probably this week. At the time of going to press, however, nothing definite was known, but when the House of Commons assembled on Tuesday, the Chancellor of the Exchequer, before moving the adjournment, announced that the Premier would make a statement on Friday. It is therefore highly probable that none of the Bills mentioned in The Hospital last week as being down for second reading will come up for consideration this year. The only questions of direct medical interest of which notice has been given are two by Sir J. D. Rees. By one of these the attention of the Secretary of State for India is directed to a memorial from the civil servants in the Punjab, representing that the substitution of Indian assistant surgeons for European civil surgeons causes hardships to families of Europeans whose work compels them to reside in civil stations. Sir J. D. Rees proposes to ask whether it is intended to pursue this policy further before an independent medical profession has been developed in India and is able to supply civil surgeons of equal qualifications with British and Irish surgeons at present available. The other question refers to the Indian traffic in opium and the use of preparations of the poppy as a medicine in India.

